# The NOTCH Pathway and Its Mutations in Mature B Cell Malignancies

**DOI:** 10.3389/fonc.2018.00550

**Published:** 2018-11-26

**Authors:** Francesca Arruga, Tiziana Vaisitti, Silvia Deaglio

**Affiliations:** ^1^Italian Institute for Genomic Medicine, Turin, Italy; ^2^Department of Medical Sciences, University of Torino, Turin, Italy

**Keywords:** NOTCH, signaling, gene mutations, B cell development, mature B cell malignancies

## Abstract

The systematic application of next-generation sequencing to large cohorts of oncologic samples has opened a Pandora's box full of known and novel genetic lesions implicated in different steps of cancer development and progression. Narrowing down to B cell malignancies, many previously unrecognized genes emerged as recurrently mutated. The challenge now is to determine how the mutation in a given gene affects the biology of the disease, paving the way to functional genomics studies. Mutations in NOTCH family members are shared by several disorders of the B series, even if with variable frequencies and mutational patterns. *In silico* predictions, revealed that mutations occurring in NOTCH receptors, despite being qualitatively different, may have similar effects on protein processing, ultimately leading to enhanced pathway activation. The discovery of mutations occurring also in downstream players, either potentiating positive signals or compromising negative regulators, indicates that multiple mechanisms in neoplastic B cells concur to activate NOTCH pathway. These findings are supported by results obtained in chronic lymphocytic leukemia and splenic marginal zone B cell lymphoma where deregulation of NOTCH signaling has been functionally characterized. The emerging picture confirms that NOTCH signaling is finely tuned in cell- and microenvironment-dependent ways. In B cell malignancies, it contributes to the regulation of proliferation, survival and migration. However, deeper biological studies are needed to pinpoint the contribution of NOTCH in the hierarchy of events driving B cells transformation, keeping in mind its role in normal B cells development. Because of its relevance in leukemia and lymphoma biology, the NOTCH pathway might represent an appealing therapeutic target: the next few years will tell whether this potential will be fulfilled.

## Introduction

Mature B cell malignancies originate from B lymphocytes that can transform at virtually every stage of the differentiation process, depending on the oncogenic events driving transformation and on accessory stimuli contributing to the accumulation and expansion of malignant B cells (1). Reliance on a combination of cell-intrinsic and -extrinsic factors to drive transformation is a shared feature among this clinically and molecularly heterogeneous group of tumors. In fact, some of the genetic mutations defining these tumors hit on signaling pathways that render neoplastic B cells independent of the environment, by driving proliferative or anti-apoptotic pathways, while other mutations target molecular components of microenvironmental signaling networks. The resulting picture is likely responsible for the profound alterations of the phenotype and functional responses of the non-neoplastic elements of the environment that become “tumor-friendly,” including T lymphocytes and macrophages (2, 3).

NOTCH pathway is one of the most evolutionarily conserved signaling cascades across species that regulates important cell fate decisions during embryonic development. Physiologically, signaling through NOTCH family members operates in a context-dependent way promoting cell proliferation, cell death and activating specific differentiation programs (4). In adult tissues, NOTCH-mediated signals are important regulators in the maintenance of self-renewal, contributing for example to myogenesis, neurogenesis and lymphocyte development (5). Considering its multiple roles in a wide range of processes and tissues, aberrations resulting in gain or loss of NOTCH signaling components and functions have been linked to a variety of disorders including solid cancers (6) and hematological malignancies (7), where NOTCH can act either as an oncogene or as a tumor suppressor. In the past decade an increasing number of reports described recurrent gain-of-function mutations of *NOTCH1* and *NOTCH2* in lymphoproliferative disorders of the B series, including chronic lymphocytic leukemia (CLL), mantle cell (MCL), splenic marginal zone (SMZL), diffuse large B cell (DLBCL) and follicular (FL), Burkitt's (BL) and Hodgkin's (HL) lymphomas. Non-mutational mechanisms of NOTCH activation have also been reported in multiple myeloma (MM) (8, 9).

This review will cover the main aspects of NOTCH contribution to B cell malignancies, starting from the mechanisms through which NOTCH signaling drives normal B lymphocyte development and commitment, in order to understand how pathway deregulation and genetic aberrations may influence B cell transformation.

## Notch pathway components and mechanisms of signaling

**Figure 1:**
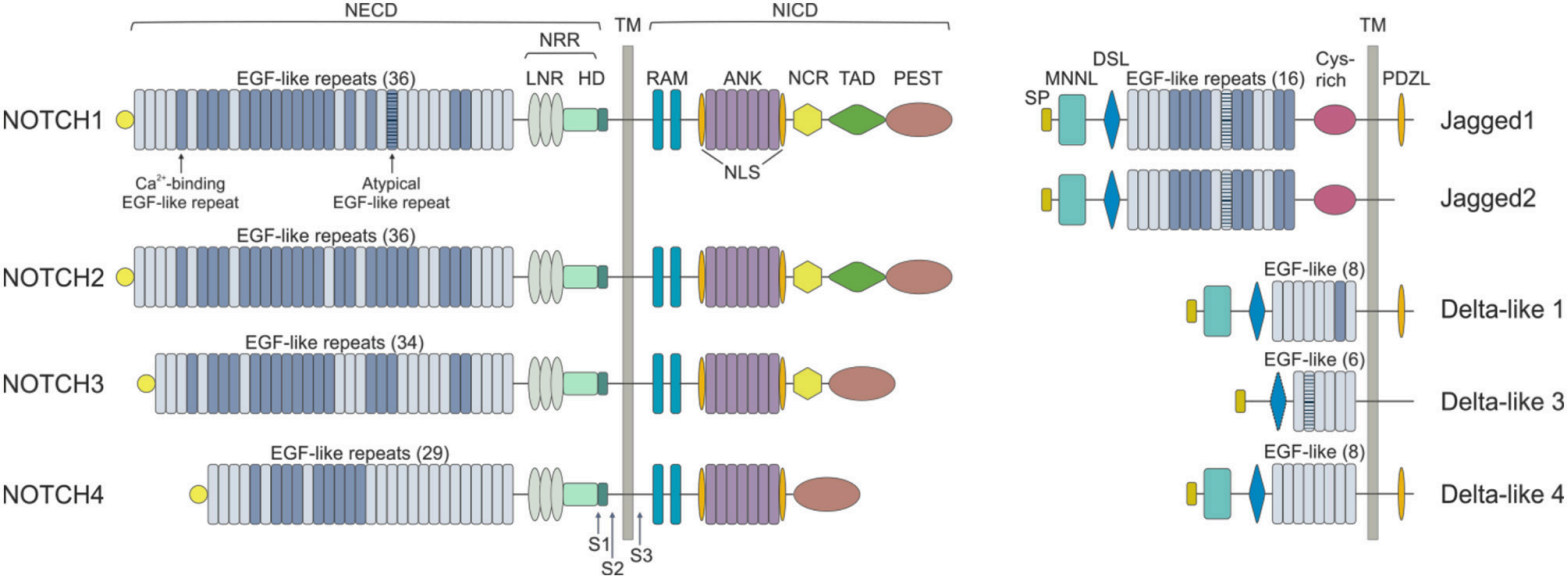
NOTCH receptors and ligands. NOTCH receptors are structurally conserved type I proteins. There are four mammalian NOTCH receptors (NOTCH1-4) that contain multiple extracellular epidermal growth factor (EGF) repeats (from 29 to 36). Specific EGF repeats mediate ligand interactions. EGF repeats are followed by the negative regulatory region (NRR), which is composed of three cysteine-rich Lin repeats (LNR) and a heterodimerization domain (HD). NOTCH also contains a transmembrane domain (TM), an RBPJk associated module (RAM) domain, a nuclear localization sequences (NLS), a seven ankyrin repeats (ANK) domain, a NOTCH cytokine response (NCR) region, a transactivation domain (TAD) and a proline-glutamic acid-serine-threonin rich (PEST) domain. Mammalian NOTCH proteins are cleaved by furin-type convertases, which convert the NOTCH polypeptide into a NOTCH extracellular domain (NECD) and NOTCH intracellular domain (NICD) heterodimer that is connected by non-covalent interactions. After ligand binding, NOTCH is cleaved by metalloproteases and γ-secretase (S1, S2, and S3). NOTCH ligands can be divided into Jagged (Jagged 1 and Jagged 2) and Delta-like (DLL1, DLL3, and DLL4) groups on the basis of their domain composition. The extracellular domain of ligands is characterized by a NOTCH ligand N-terminal domain (MNNL), a Delta/Serrate/LAG-2 (DSL) domain, EGF motifs and a cysteine-rich (Cys) domain. DLLs lack this latter domain. The intracellular portion may contain a post-synaptic density protein ligand domain (PDZL).

Mammals express four NOTCH receptors (NOTCH1-4), each encoded by a different gene, that interact with five different ligands (DLL1,-3,-4 belonging to the Delta-like family and Jagged1 and−2 which are part of the Serrate family of ligands) (10) (Figure 1). NOTCH receptors are single-pass type I transmembrane proteins showing high structure homology (especially NOTCH1 and NOTCH2) and displaying both common and unique functions. They are synthesized as single precursors that maturate in the Golgi apparatus upon proteolytic cleavage (S1) by a furin-like convertase. Mature receptors expressed on the cell surface are heterodimers composed by an N-terminal extracellular region (EC) non-covalently associated with a transmembrane (TM) domain and a C-terminal intracellular (IC) subunit (11). The EC portion of NOTCH receptors contains a series of epidermal growth factor (EGF)-like repeats (29–36), some of which are crucial in mediating ligand interactions and responses (12). Within the EC domain, the EGF-like repeats are followed by a juxtamembrane negative regulatory region (NRR), which contains three Lin12/Notch repeats (LNRs) and a heterdimerization domain (HD), and which prevents NOTCH activation in the absence of ligands. The IC portion of the receptors consists in a protein-binding RBPJk-associated molecule (RAM), seven ankyrin repeats, and less conserved regions including a transcriptional activation domain (TAD) and a C-terminal region rich in proline, glutamate, serine and threonine (PEST domain), which regulates protein stability and degradation as it contains the substrate site that is recognized by E3 ubiquitin ligases (*degron* domain) (10, 13). Among family members, NOTCH1 and –2 are the most widely expressed receptors, being present in many tissues at the developmental stage, as well as in adults, while NOTCH3 is found mainly in vascular smooth muscle and pericytes, and NOTCH4 is most highly expressed in endothelium (6).

NOTCH ligands are also type I TM proteins showing high structural homology within the Delta-like and Serrate families, but their expression patterns are less well characterized than those of the receptors (4) (Figure 1). The strength and outcome of receptor-ligand interactions are modulated by post-translational modifications of NOTCH receptors. The EGF repeats of NOTCH EC region can be modified by the addition of *O*-fucose and *O*-glucose residues, which can in turn lead to further modifications (14). The addition of *O*-fucose by protein *O*-fucosyltransferase 1 (Pofut1) is required for subsequent glycosylation of NOTCH receptors by the Fringe family of glycosyltransferases (Lunatic, Manic and Radical Fringe). Glycolsylation potentiates the interactions of NOTCH receptors with DLLs, while reducing responsiveness to JAGs (15).

**Figure 2:**
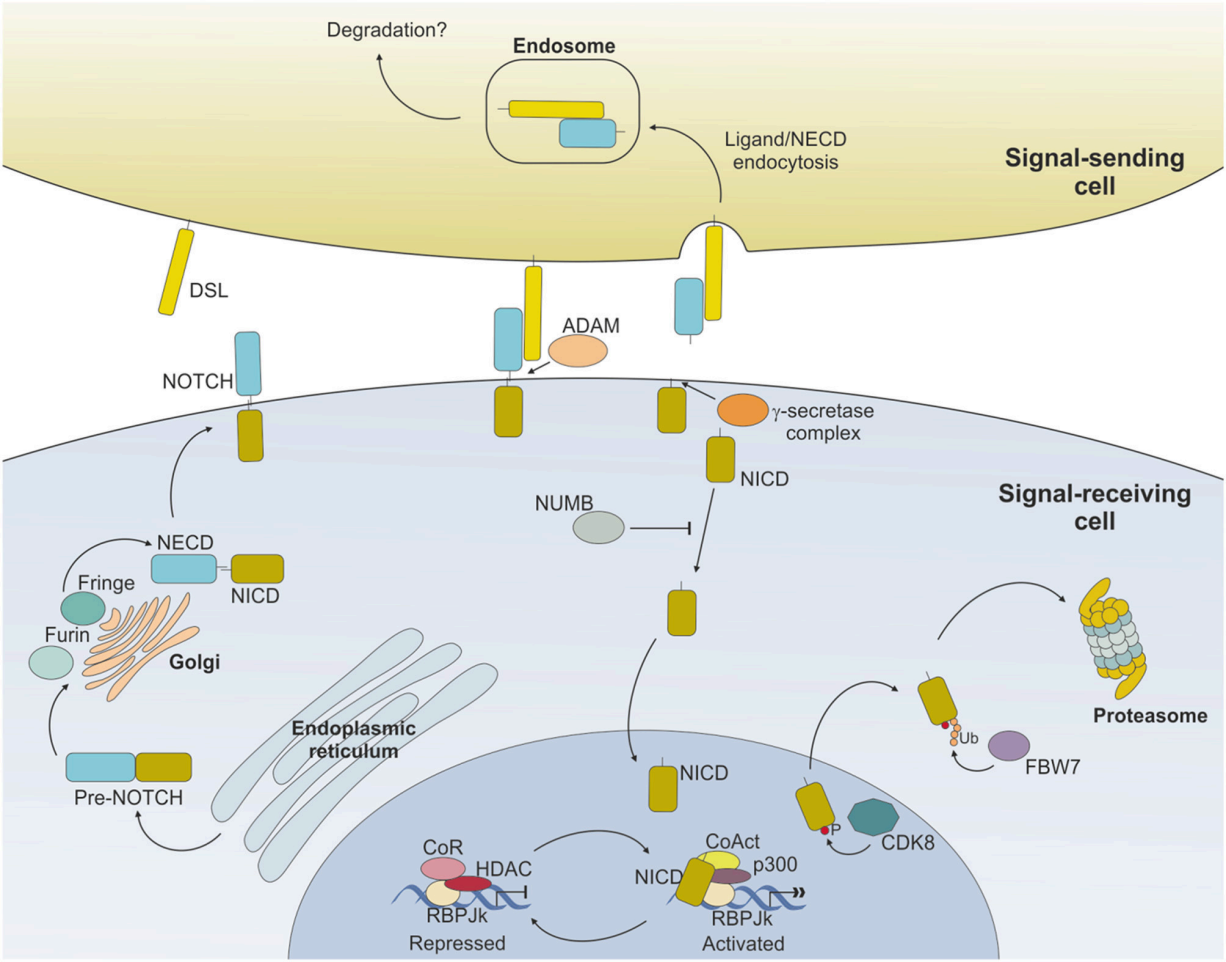
NOTCH signaling pathway. Ligand expressed on the surface of the signal-sending cell binds to NOTCH expressed on the surface of the signal-receiving cell and induces sequential cleavages by A-Disintengrin-And-Metalloprotease (ADAM) and γ-secretase, ultimately releasing the NOTCH intracellular domain (NICD) from the membrane. NICD translocates to the nucleus where it mediates the displacement of co-repressors (CoR) and Histone DeAcetylase Complex (HDAC) and directly interacts with RBPJk recruiting co-activators (CoAct), finally inducing target gene transcription. The signaling pathway is shut down by phosphorylation of the NICD subunit by C/Cyclin-dependent kinase 8 (CDK8) and subsequently poly-ubiquitinated by F-box containing protein (FBW7) and degraded via proteasome. The strength and outcome of receptor-ligand binding can be modulated by post-translational modifications of NOTCH receptors, operated by the Fringe family of enzymes.

Binding of NOTCH to a ligand on neighboring cells leads to a conformational change of the receptor and rescues the inhibition imposed by the NRR, exposing a cleavage site (S2) for ADAM metalloproteases close to the TM domain (16) (Figure 2). ADAM-mediated cleavage releases the TM-IC regions from the EC portion of the receptor and this effect is facilitated by, and partly depends on, a mechanical force delivered to the receptor by the signal-sending cell through ligand endocytosis (17). S2 cleavage generates a short-lived membrane-bound form of NOTCH (N_EXT_, NOTCH extracellular truncated) that is rapidly further cleaved by the γ-secretase complex (S3), releasing NOTCH IC domain (NICD) from the membrane and allowing its translocation to the nucleus (18, 19). Once in the nucleus, the NICD forms a transcriptional complex, having RBPJk as core DNA-binding factor. By binding the transcription factor, NICD alters the composition of RBPJk-tethered complexes, shifting the composition from that of a repressor to an activator of transcription. Specifically, NICD displaces co-repressor molecules bound to RBPJk, such as HDACs, SHARP, MINT and SPEN (11, 20–22) and recruits transcription co-activators of the Mastermind-like family [MAML, (23)], as well as the histone acetyl transferase p300 (24) and the histone demethylase KDM1A [also known as LSD1, (25)], to initiate transcription of NOTCH target genes. Major NOTCH target genes include the basic helix-loop-helic (bHLH) class of transcription factors, such as *HES1* and *HEY1* (26), which act as repressor of transcription playing critical roles in developmental processes, as well as in transformed cells. Furthermore, NOTCH activity fosters a self-regulating feedback loop by transcriptionally controlling genes encoding cytoplasmic protein that modulate NICD translocation to the nucleus or binding to RBPJk, such as *Deltex1* (*DTX1*) and *NRARP* (27, 28). Among the genes under a direct NOTCH-dependent transcriptional control, *MYC* is an important mediator of NOTCH effects in the transformation process for several tumor types.

Activity of NOTCH signaling is then terminated through the phosphorylation of the PEST domain by cyclin C/cyclin-dependent kinase 8 (CDK) 8 and the subsequent ubiquitinylation by the E3 ligase complex, containing the F-box protein FBW7, which drives NICD to proteasome-mediated degradation (29–31) (Figure 2).

## Notch signaling participates in B cell development

In the study of normal immune system, the role of NOTCH has been mostly characterized in T cell development, where it regulates T cell commitment of common lymphoid progenitors (CLPs) at the expenses of B cell differentiation (32). B lymphopoiesis takes place in the bone marrow (BM) where newly formed B cells are generated from CLPs, before branching into a B1 subset or continuing toward pro-B cells. The former gives rise to long-lived lymphocytes, predominantly found during fetal and neonatal life, and residing in the peritoneal and pleural cavities in adults, where they complete their differentiation into IgM-producing plasma cells (33). On the contrary, pro-B cells differentiate to immature B lymphocytes that migrate to the spleen as transitional type 1 (T1) B cells, then developing into type 2 transitional B cells (T2). T2 lymphocytes can further differentiate in mature B2 cells, which represent the predominant B population in adult secondary lymphoid organs and the main effectors of adaptive immunity. Following specific microenvironmental signals, they differentiate in two main subsets in a T cell-independent or -dependent way, respectively: (i) marginal zone (MZ) B lymphocytes, residing exclusively in the spleen, and (ii) follicular (FO) B cells that can circulate and are more widely distributed in splenic and lymph node (LN) follicles, as well as in the BM, and can participate in germinal center (GC) reaction (34).

The expression and functions of NOTCH components in normal B cell biology has so far proven controversial for many aspects, likely because these proteins show distinct roles in early *vs*. late phases of B cell development and when considering fetal or adult B cell generation. For instance, in a study performed on human fetal B cells, Bertrand and colleagues showed that NOTCH1 mRNA and proteins are expressed, throughout normal B cell development, whereas NOTCH2 expression is limited to late pre-B cells expressing low levels of surface immunoglobulin. The authors hypothesized that, given its ubiquitous expression in B cell development, NOTCH1 could modulate proliferation and B cell differentiation through multiple developmental checkpoints (35). In contrast to these observations, other studies examining adult murine B cell subsets, described a modulation of NOTCH components through B cell development, with NOTCH1 and NOTCH3 being highly expressed in pro-B cells, progressively decreasing in pre- and immature B cells and maintaining low levels in peripheral B cells. At variance, NOTCH2 transcript was progressively upregulated with sustained expression in peripheral B cells (36).

Ligands belonging to both DLL and Jagged families are expressed on the surface of a wide range of BM stromal cells and can exert different effects on signal-receiving cells depending on the differentiation status of B cell progenitors and the cooperation with accessory stimuli present in the niches (37). Importantly, their expression is discontinuous, turning NOTCH signaling on or off according to developmental needs. B cell development is driven by specific cytokines such as CXCL12 in the earliest phases and interleukin (IL)-7 as differentiation proceeds (38). Early B cell progenitors such as pre-pro B cells reside in microenvironmental niches where CXCL12 is expressed, while NOTCH ligands are not. In fact, CLPs commitment to the B cell lineage requires NOTCH signaling shut down, at variance with T cell development, which is strictly dependent on NOTCH1 expression (39). As development proceeds, B cell precursors migrate from CXCL12- to IL-7-expressing stroma, an environment where both DLL and Jagged ligands are expressed, suggesting that NOTCH signaling may have a role in committed B cells developing in the BM and ready to migrate toward secondary lymphoid niches to complete the differentiation process (37, 40).

Furthermore, beside BM stroma, developing B cells can themselves surface express NOTCH ligands, thereby suggesting that NOTCH signaling can operate through multiple cellular interactions, other than stromal cells. Indeed, Bertrand and colleagues observed that Jagged-1 is mainly expressed by BM stromal cells whereas Delta-like ligands are preferentially expressed in pro-B and pre-B cells, hypothesizing different outcomes of NOTCH signaling according to different ligand binding. They suggested that NOTCH-DLL interactions occurring between B cell precursors with equivalent developmental potential (termed “lateral signaling”), may contribute to the maintenance of a normal B lineage homeostasis, by signaling some cells to commit to a specific differentiation fate, while maintaining others in the original precursor state (35).

While the impact of NOTCH signaling in early B cell development is still controversial and only partially clarified, its role later on in the differentiation process is better documented. For example, conditional knock out of NOTCH2 in murine B cells results in the complete absence of MZ B cells, without affecting other B cell subsets, and in increased mortality due to blood-borne bacterial infections (36, 41). Expression of NOTCH2 is thus essential to drive B cells toward MZ maturation. Furthermore, NOTCH ligands show partially overlapping expression patterns in the spleen, with preferential association with vascular stromal cells. Specifically, stromal cells within the MZ strongly express DLL1, which is needed for complete MZ B cell development (42, 43).

**Figure 3:**
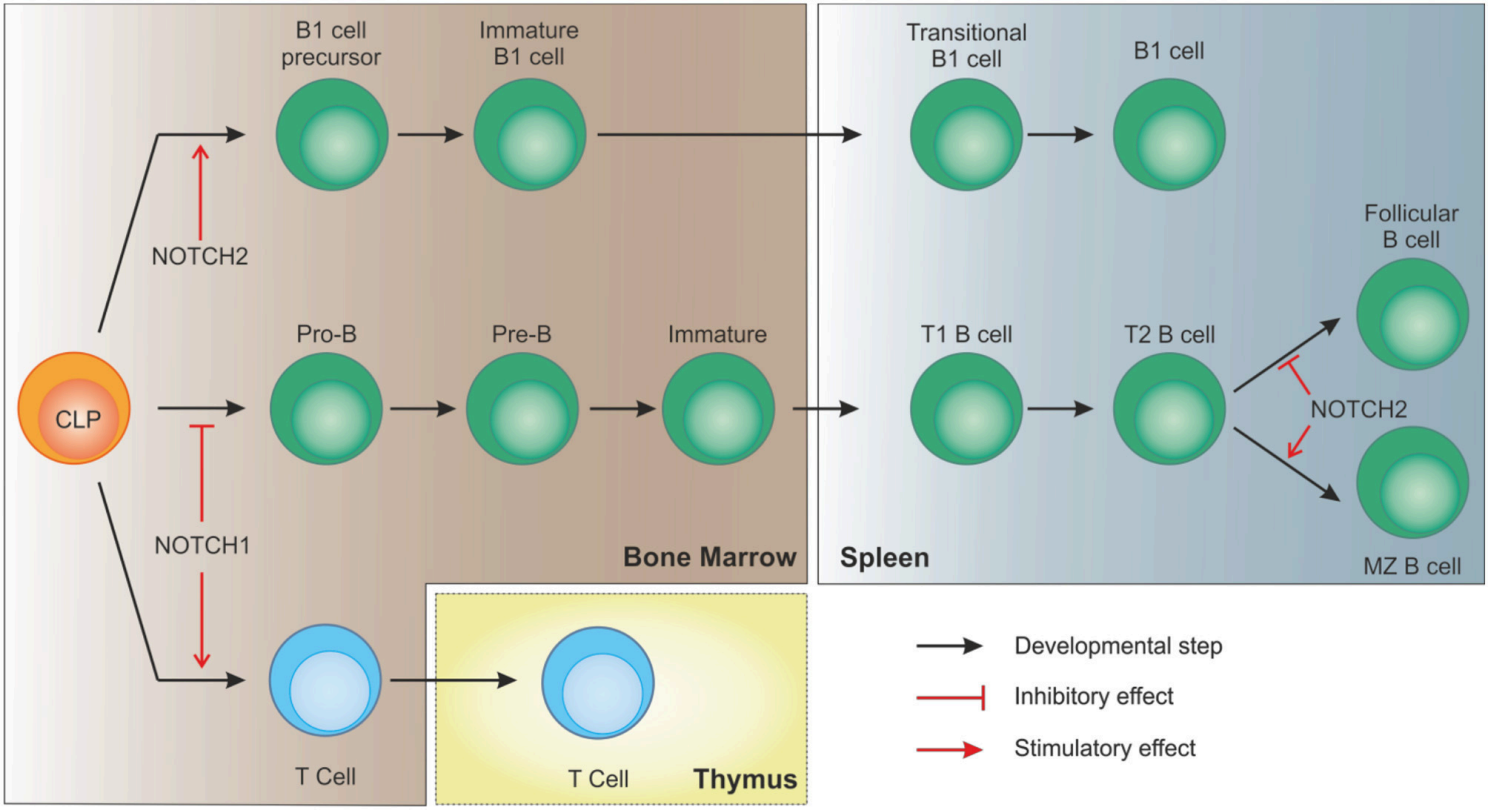
NOTCH signaling in B cell development. In bone marrow-residing CLPs, NOTCH1 signaling must be switched off to allow proper B cell development. On the contrary, after migration of immature B cells to the spleen, interaction of DLL1 with NOTCH2, with the involvement of recombinant signal binding protein for immunoglobulin kJ region (RBPJk), induces NOTCH signaling in transitional B (T2) cells to specify marginal zone B cells, as opposed to follicular B cells. NOTCH2 signaling is also necessary for CLPs to differentiate toward B1 cells.

Together with MZ cells, B1 lymphocytes are considered innate-like lymphocytes showing an antibody repertoire to recognize pathogen-associated molecular patterns. B1 cells can originate from early lymphoid progenitors and CLPs but not from pro-B cells, indicating that cell fate of B progenitors becomes restricted as soon as cells show commitment toward a specific B lineage (44). Similar to B2 MZ differentiation, NOTCH2 signaling takes part in B1 cells development and maintenance. As shown in a study by Witt and colleagues, NOTCH2 haploinsufficiency resulted in reduced B1 B cells in the peritoneal cavity compared to wild-type mice (45). Interestingly, the same group also showed that, at variance with NOTCH1 activation in CLPs that drives T cell commitment, ectopic induction of NOTCH2 signaling, obtained by transducing BM cells with the activated intracellular domain of NOTCH2 (ICN2), boosted early B cell development and B1 commitment while blocking B2 progression at the pre-B stage (46). These observations suggest that, despite the strong similarities in signaling components, activation of NOTCH pathway plays a critical role in lymphocyte development showing distinct non-redundant functions (Figure 3).

Finally, NOTCH signaling also contributes to B cell activation and terminal differentiation to antibody-secreting cells (ASCs) by acting synergistically with B cell receptor (BCR) pathway and co-stimulatory signals such as CD40 or BAFF. The outcome for B cells is increased cell survival and proliferation, along with activation and antibody production (47, 48).

Given the multiple implications of NOTCH signaling in B cell development, it is perhaps not surprising that alterations of pathway activity are associated with B cell malignancies. The following paragraphs of this review will focus on the deregulation of NOTCH pathway in various models of B cell neoplasia, starting from chronic lymphocytic leukemia where the biological meaning of this pathway in the pathophysiology of the disease is better characterized than in other B cell malignancies.

## NOTCH1 is aberrantly activated in chronic lymphocytic leukemia (CLL)

Among B cell malignancies, CLL represents the most frequent adult leukemia with an incidence rate of ~3.9/100,000/year (49). It is characterized by the progressive accumulation of mature-looking CD5^+^/CD23^+^ B lymphocytes in the peripheral blood (PB) with infiltration of lymphoid tissues such as spleen and LNs (50). Clinically, disease course is highly heterogeneous in terms of presentation, outcome and therapy responses, with patients either showing an indolent disease with a limited impact on life expectancy or exhibiting a rapidly progressive disease despite early treatment initiation.

**Figure 4:**
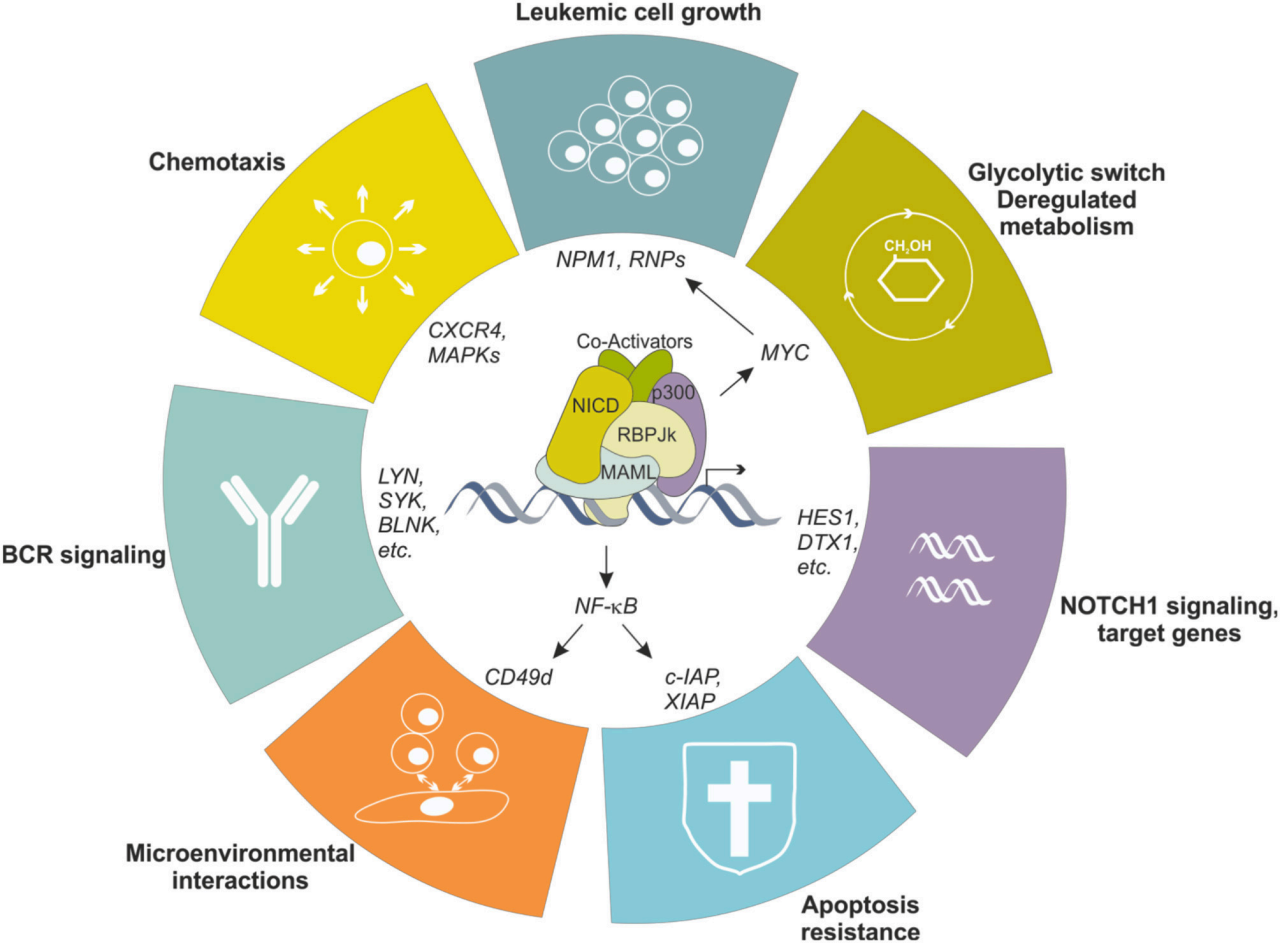
NOTCH1 transcriptional effects in CLL cells. NOTCH1 signaling is aberrantly activated in CLL cells, resulting in transcriptional regulation of several genes, in turn impacting different biological aspects of the neoplastic cells. Increased NOTCH1 activity contributes to leukemic cell growth, protection from apoptosis, metabolic switching toward a glycolytic metabolism, increased migration in response to specific chemokines and facilitates interactions with microenvironment. Moreover, it upregulates several genes encoding proteins insisting on the BCR signaling pathway, the driving force for CLL cells.

To simplify, this largely depends on the B cell precursor CLL originates from, which is driven by antigen-mediated triggering of BCR together with multiple accessory signals, provided by cell-bound and soluble factors, that reinforce survival and proliferative advantage of leukemic cells (51, 52). Among others, CLL cells show overexpression and aberrant activation of NOTCH1 (53, 54), and this is markedly evident in lymphoid niches (55), likely due to the fact that stromal cells in the BM and in LNs strongly express NOTCH ligands (56, 57). Leukemic cells themselves express NOTCH ligands on the surface, suggesting the existence of autologous signaling (57). Increased activity of NOTCH1 pathway protects leukemic cells from apoptosis through multiple mechanisms, among which the crosstalk with NF-κB signaling represents a key feature in CLL biology, as it directly regulates expression of anti-apoptotic genes (e.g., c-IAP2 and XIAP) and surface molecules (e.g., CD49d) that facilitate interactions with microenvironment, feeding a pro-leukemic loop (58) (Figure 4). Moreover, NOTCH1 promotes CLL cell growth and active proliferation by upregulating genes related to ribosome biogenesis and protein translation such as *NPM1* and ribosomal proteins (*RNPs*) (59). Even if a formal demonstration is still missing, these effects are likely mediated through the up-regulation of MYC, as also supported by the involvement of a NOTCH1-MYC axis in the glycolytic switch induced in CLL cells by stromal cells, an event contributing to stroma-mediated chemoresistance (60). Finally, NOTCH1 transcriptional activity in CLL upregulates genes insisting on BCR and cytokine/chemokine signaling, therefore further sustaining leukemic cell survival and proliferation possibly amplifying BCR-mediated effects through a synergistic cooperation (48, 54) (Figure 4).

A further indication underlining the importance of NOTCH1 signaling in CLL comes from the finding that mutations in this gene have emerged as one of the most frequent single gene alterations found in CLL at diagnosis (5–15% of cases) (61). Prevalence increases to ~20% when considering chemorefractory patients, and up to ~30% in CLL transforming to aggressive lymphoma (Richter Syndrome, discussed below) (62). The majority of *NOTCH1* mutations in CLL occurs within the last exon of the gene and affects the C-terminal portion of the receptor. Specifically, the mutation accounting for ~80% of cases is a 2-bp deletion in exon 34 that shifts the reading frame (c7541_7542delCT) and generates a premature stop codon (P2514fs^*^4), truncating the PEST domain (61–63). Frameshift mutations affecting different nucleotides in exon 34 (64), as well as mutations in the non-coding 3′ untranslated region (3′-UTR) of *NOTCH1*, favoring alternative splicing events with a cryptic donor site in exon 34 (65), have also been described with similar effects on the protein. Mechanistically, PEST domain truncation leads to the loss of the *degron* domains that direct NOTCH1 to proteasomal degradation, therefore affecting the physiological turnover of NICD, increasing its stability and prolonging pathway activity (66). At variance with T-ALL where mutations in NOTCH1 HD facilitate ligand-independent activation (67), triggering of NOTCH1 signaling in CLL strictly relies on the interaction with the ligand(s), even in the presence of PEST mutations, as these lesions result in the stabilization of ligand-triggered cleaved NOTCH1 rather than in autonomous signaling activation (57).

Clinically, *NOTCH1* mutations identify patients with a worse prognosis in terms of therapy responses and with higher risk of disease transformation into an aggressive lymphoma (68). *NOTCH1-*mutated samples are enriched in chemorefractory CLL and *in vitro* evidence shows a marked resistance to fludarabine-induced apoptosis, which can be rescued by NOTCH1 inhibitors (57, 69). More recently, an association between *NOTCH1* mutations and reduced benefits from anti-CD20-based chemoimmunotherapy regimens was described (70), likely as a consequence of downmodulation of surface CD20 in this patient subset compared to WT samples (71). From the biological standpoint, reduced CD20 expression is due to the fact that accumulation of mutated NICD in the nucleus perturbs a delicate balance between nuclear interactors, ultimately impacting on the amount of free HDAC that can bind to and silence other genomic regions, including the CD20 promoter (71). Understanding this mechanism prompted the idea that NOTCH1 may exert its effects not only through a direct transcriptional regulation of gene expression, but also indirectly, by altering epigenetic regulation. We recently demonstrated that *NOTCH1*-mutated cells have an increased migratory potential in response to chemokines that regulate homing of CLL cells to lymphoid niches, such as CCL19, because of a higher expression of the chemokine receptor CCR7. The underlying mechanism is that free HDAC, displaced from the RBPJk-tethered complex by an excess of NICD, can interact with and stabilize DNMT3A, which in turn suppresses the expression of the tumor suppressor gene *DUSP22*. Loss of DUSP22 phosphatase activity leads to aberrant activation of MAPK and STAT3 signaling, both crucial in CLL homeostasis as downstream players of growth and chemokine receptors, and consequently to a STAT3-dependent upregulation of CCR7 expression and responsiveness (66). Conceivably, *NOTCH1*-mutated CLL cells may be more prone to reach privileged lymphoid niches that provide pro-leukemic stimuli including NOTCH1 ligands, thereby further fueling pathway activation.

At diagnosis, *NOTCH1* mutations can be found either as a clonal defect, present in the large majority of leukemic population, or at the subclonal level (cut-off of Variance Allele Frequency < 12%), suggesting that they might be acquired at different steps during CLL development. Specifically, clonally represented mutations are indicative of an early event, whereas subclonal mutations are thought to be acquired later and to be progressively selected. In favor of the view that the acquisition of NOTCH1 mutations is a relatively late event, is the observation that they might be subsequent to at least one driver alteration such as a chromosomal aberration. Accordingly, *NOTCH1* mutations are strongly associated with trisomy 12 (72). On the other hand, *NOTCH1* mutated subclones have been detected in high-count monoclonal B lymphocytosis (MBL), considered a pre-malignant state potentially evolving to CLL (73, 74). In line with the idea that NOTCH1 alterations may be involved in CLL initiation, several papers revealed the presence of *NOTCH1* mutations in early hematopoietic progenitors of CLL patients harboring the defect at the time of disease presentation (75, 76). From the clinical standpoint, the prognostic impact of a low *NOTCH1* mutated burden, as well as the evolution of the mutated CLL clone during disease progression, remains unclear. Some studies reported that subclonal *NOTCH1* mutations are not associated with chemorefractory disease and remain stable through the follow-up of the disease, suggesting that they may not confer growth advantage to leukemic cells over WT cells, thereby not being prognosticators of aggressive/progressive CLL (77, 78). In contrast, other groups observed that CLL patients harboring *NOTCH1* mutations in a small portion of the leukemic population show a shorter time to first treatment and reduced overall survival similar to that of clonally mutated CLL (72, 79). Discrepancies in clinical observations may be due to differences in the cohorts under examination. In the sample cohort investigated by Rasi and colleagues, *NOTCH1* mutations were mainly present already at the clonal level, with only few patients showing a mutated fraction below the limit of Sanger sensitivity, likely not fully recapitulating the dynamics of disease evolution in which subclonal *NOTCH1* mutations might be implicated (78).

Independently of whether aberrant NOTCH1 signaling is the result of genetic alterations or of a permissive environment, this pathway plays a critical role in CLL pathogenesis and progression and could represent a suitable therapeutic target.

## Deregulated notchs in mature B cell malignancies other than CLL

**Figure 5:**
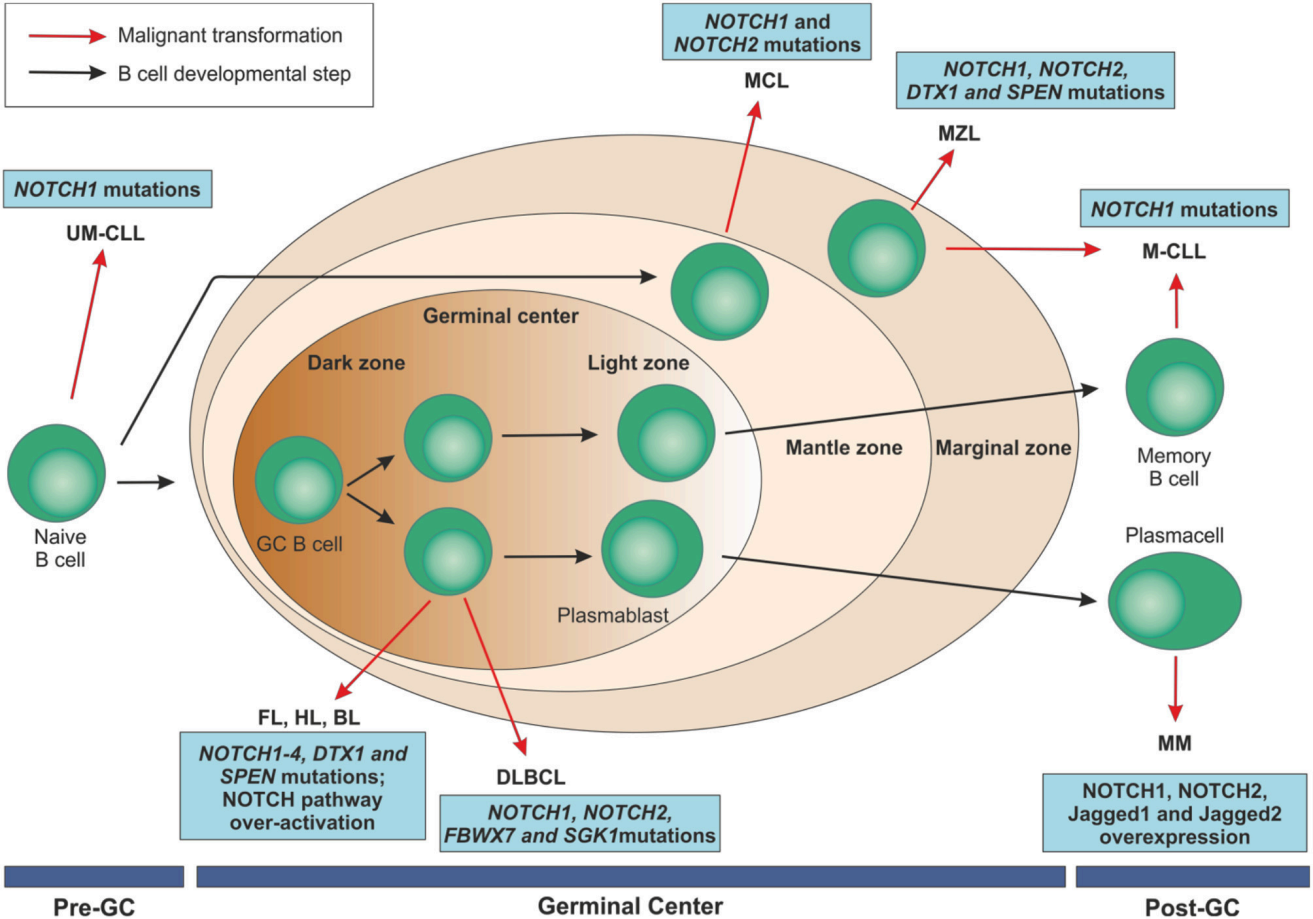
Mature B cell malignancies classification according to their normal counterpart and overview of NOTCH implications in these disorders. After antigen contact, naïve B cells are activated and enter the germinal centers. Maturation through a T cell dependent process leads to centroblast differentiation in the dark zone with high proliferation rate and somatic mutations of immunoglobulin variable regions and continue their maturation through the light zone. After that, they can become memory B cells or terminally differentiated plasmacells. On the contrary, transitional B cells are selected into a mature B cell repertoire in a T cell-independent way through the marginal zone of the spleen. B cell malignancies can arise from B lymphocytes at each maturation step. NOTCH family members have been identified as recurrently mutated in some of these disorders or to be over-expressed, ultimately resulting in an aberrant activation of the pathway.UM-CLL, IGVH unmutated chronic lymphocytic leukemia; M-CLL, mutated chronic lymphocytic leukemia; FL, follicular lymphoma; HL, Hodgkin's lymphoma; BL, Burkitt's lymphoma; DLBCL, diffuse large B cell lymphoma; MCL, mantle cell lymphoma; MZL, marginal zone lymphoma; MM, multiple myeloma; GC, germinal center.

A role for NOTCH in tumor development, progression, and drug resistance has also been highlighted in B cell malignancies other than CLL. For example, NOTCH deregulation was described in HL, BL (80, 81), DLBCL (82, 83), and MM (84, 85) (Figure 5).

For most of them, the current knowledge derives from the massive unbiased sequencing approaches on large cohorts of samples that highlighted recurrent mutations in genes of the NOTCH pathway, sharing mechanistic effects on the protein and pathway activity. MM represents an exception, as deregulation of NOTCH relies mostly on the overexpression of signaling components. Overall, form the functional standpoint, the role of NOTCH alterations in these B cell malignancies is less well characterized than in CLL. The following sections of this review are intended to explore the expression, genetics and role of NOTCH family members in these diseases.

### Mantle cell lymphoma (MCL)

MCL is an aggressive mature CD5^+^/CD23^−^ B cell malignancy, arising from naïve pre-germinal center B cells of the inner mantle zone and accounting for 6–7% of non-Hodgkin lymphomas (86). The clinical history is highly variable and ranges from indolent forms to a very aggressive disease with a short median survival, frequent relapses and increasing resistance to chemotherapy (87, 88). Regarding the pathogenesis, the primary alteration in MCL is the translocation t(11;14)(q13;q32), that leads to the juxtaposition of the *CCDN1* gene with the *IGH* locus, resulting in cyclin D1 overexpression and constitutive activation with consequent cell cycle deregulation (89, 90). Beside this lesion, secondary genetic alterations are needed to induce lymphomagenesis by disrupting additional critical pathways. Several papers described alterations involving *CDKN2A, CDKN2B, TP53, RB1* resulting in deregulation of MCL cell proliferation (89–92). In order to further understand the biology of MCL, discover novel pathogenic lesions and potentially identify novel targets for therapy, Kridel and colleagues exploited a powerful whole transcriptome shotgun sequencing (RNAseq) approach applied to primary samples and cell lines (93). Along with mutations in genes known to be involved in MCL pathogenesis, they found recurrent mutations in *NOTCH1* (12% for primary samples and 20% in cell lines), the majority located in exon 34 that encodes the PEST domain, and thus resulting in an abnormally over-activation of NOTCH1 signaling pathway. The mutational pattern (non-sense truncating mutations and small frame-shift indels) and frequency were similar to what described in CLL (61, 62). Mutations in *NOTCH1* were associated with a shorter overall survival, suggesting a negative prognostic role for *NOTCH1* in MCL. Functionally, Kridel and colleagues found that MCL cell lines were sensitive to NOTCH1 inhibition, as indicated by reduced proliferation and induction of apoptosis, as well as by the modulation of a specific gene expression profile (93). In line with these findings, it was recently demonstrated that NOTCH signaling regulates, directly or indirectly through MYC, a gene signature insisting on BCR signaling, RNA metabolism, and chromatin/transcriptional regulation, thus providing a potential basis for the selective drive of NOTCH gain-of-function mutations in MCL (94). These results have broad implications in B cell lymphomagenesis and pave the way for developing novel therapeutic strategies involving the use of NOTCH pathway inhibitors in these cancers.

By using whole-genome and whole-exome sequencing analysis applied to a large cohort of MCL patients, Bea and colleagues reported that also *NOTCH2* mutations are present as an alternative and mutually exclusive phenomenon to *NOTCH1* alterations in aggressive tumors with a poor prognosis (95). The mutation pattern is similar to that reported for *NOTCH1*, with the generation of a premature stop codon within the PEST domain. Accordingly, gene expression analysis highlighted a different profile, with *NOTCH2* mutated cases displaying an up-regulation of genes involved in cell-cycle and metabolic pathways, together with genes directly regulated by NOTCH2 (95).

### Follicular (FL), hodgkin's (HL), and burkitt's (BL) lymphomas

Mature follicular B cells represent the pool of recirculating peripheral lymphocytes that generate both plasmablasts and memory cells in response to pathogens. FL arises from germinal center B cells blocked in their capacity to differentiate further (96, 97). FL is the most common indolent and slowly progressive lymphoma, with a median survival of 10–14 years (98, 99). Histologic transformation to an aggressive lymphoma occurs in 2–3% of patients/year and it is associated with chemoresistance, progression and increased mortality (100). From the molecular standpoint, FLs are almost universally characterized by the t(14;18)(q32;q21) translocation which leads to a fusion of *BLC2* to regulatory elements of the *IGH* locus (101). The resulting constitutive overexpression of BCL2 abrogates the default germinal center apoptosis, likely representing an initiating oncogenic “hit.” In addition, the use of high-throughput sequencing technologies has been helpful in identifying other recurrently mutated genes that drive transformations (102). They belong to the BCR/NF-κB signaling, apoptosis, chromatin remodeling and B cell development pathways (103–108). Most of them have been functionally validated exploiting mouse models, confirming their pathophysiological role in FL and leading to the development of a new wave of drugs (109–112). Recently, by analyzing a cohort of 112 FL cases by Sanger sequencing, Karube and colleagues reported mutations also in *NOTCH1* and *NOTCH2*. For the former gene, the only detected mutation was the p.P2514fs^*^4, with no alterations in the heterodimerization domain. For *NOTCH2*, a nonsense (p.R2400^*^) and a frameshift (p.I2304fs^*^9) mutations were reported (113), resembling the profile found in SMZL (114). All these mutations lead to the truncation of the PEST domain and thus to a persistently active NICD, in line with mechanisms described for other B cell malignancies. Similar results, both in terms of frequency and pattern of mutations, were confirmed in an independent cohort by Krysiak and colleagues (115). These authors found novel mutations in *NOTCH3* (4.8%) and *NOTCH4* (4.2%), as well as in the NOTCH signaling regulators *DTX1* (5.7%) and *SPEN* (2.9%).

The finding that genes encoding NOTCH components are altered in subgroups of FLs is in apparent contrast with the physiological role that NOTCH plays in promoting maturation of B cells to MZ (see also Figure 3) (36, 41, 116, 117). On the other hand, a recent paper pointed out that NOTCH2 downmodulation is essential for FL cell survival, calling back into question the significance of NOTCH signaling in this disease (118), and suggesting that more work is needed to solve this apparent controversy.

Overall, these findings highlight the NOTCH pathway as heavily mutated in this disease, but leave open its functional role.

Overactivation of NOTCH signaling pathway has been reported also in Hodgkin's and Burkitt's lymphomas, both deriving from germinal center B cells. In the former case, tumor cells are characterized by a higher expression of NOTCH1, NOTCH2 and Jagged2, but also of Mastermind-like 2 (MAML2), an essential NOTCH co-activator (119). The final result is an over-activation of NOTCH signaling pathway as an alternative mechanism to the cell-autonomous NOTCH activity, typical of these cancer cells, as shown by the gene-expression profile studies (120). The critical role played by this pathway is underlined by the evidence that targeting of MAML proteins resulted in inhibition of NOTCH and in a reduced proliferation and growth of HL cells (119). It has also been reported that NOTCH activation can trigger NF-kB signaling, promoting survival of HL cells in cooperation with the Epstein-Barr virus (EBV) (121).

A synergistic mechanism between NOTCH and another partner has been suggested also in BL, where the counterpart is represented by the BCR, with c-myc as potential point of convergence. This cross-talk results in the modulation of proliferation and apoptosis of lymphoma cells and can be reverted by using a gamma-secretase inhibitor (80). More recently, Cao and colleagues, highlighted that specific elements present in the vascular niche contribute to the modulation of NOTCH ligand expression, specifically Jagged1, on endothelial cells that in turns activates NOTCH2 signaling in BL cells, enforcing their aggressiveness in terms of extra-nodal invasion and chemoresistance (122).

### Diffuse large B cell lymphoma (DLBCL) and richter syndrome (RS)

DLBCL is a highly heterogeneous neoplasm category (123), accounting for 30–40% of newly diagnosed non-Hodgkin lymphomas (NHL) (124).

Historically, DLBCL was thought to involve recurrent translocations of the *IGH* gene and the deregulation of rearranged oncogenes, including *BCL2, BCL6*, or *MYC*. More recently, the molecular heterogeneity of DLBCLs has been deciphered by gene expression profiling, allowing the classification into two main molecular subtypes: the germinal center B cell like (GCB) and the activated B cell like (ABC). These subtypes arise from distinct B cells at separate stages of differentiation and maturation (125), leading to well-defined gene expression profiles and different clinical outcomes and responses to immunochemotherapy, with ABC type being the most aggressive and characterized by a poor clinical outcome (126, 127).

The most frequently (≥10% of all samples) mutated genes in DLBCL belong to pathways controlling cell cycle, DNA damage response, chromatin remodeling, BCR and TLR signaling (125, 128–133) Additional genes were found to be mutated in a lower percentage of samples, but potentially relevant to DLBCL biology, including genes belonging to the NOTCH family.

Very recently, a comprehensive analysis of 304 primary DLBCLs samples integrated low-frequency alterations, recurrent mutations, somatic copy number alterations, and structural variants, to identify five groups of patients with outcome-associated coordinated genetic signatures, three of which previously undescribed. PEST mutations in *NOTCH2* and truncating alterations of its negative regulator *SPEN*, together with *BCL6* structural variants and mutations of the NF-kB pathway were associated to the Cluster 1 (C1) DLBCLs. This subset is characterized by an increased transcriptional abundance of NOTCH2 and BCL6 target genes, as highlighted by gene set enrichment analysis (GSEA). The majority of C1 DLBCL were classified as ABC-type tumors, by transcriptional profiling. This novel classification may help tailor treatment strategies in these genetically distinct DLBCL subsets (134, 135). These results built on previous findings obtained in large cohorts of lymphoma samples describing mutations in NOTCH pathway as a recurrent defect in DLBCL (83, 136–138). Although belonging to this lymphoma category, RS is considered a distinct subset of DLBCL, as it evolves from a pre-existing CLL undergoing a transformation into an aggressive lymphoma in the 10–12% of cases (123, 139). Analysis of *IGH* rearrangements indicate whether RS is clonally related or unrelated to CLL, thus if it derives from the same B cell clone or if it arises as a secondary independent neoplasia (140). This distinction is clinically relevant as clonally-related RS patients are characterized by a poorer outcome, with no response to standard therapy and with a shorter overall survival compared to clonally unrelated samples (140–142). Whole exome sequencing (WES) and copy-number analyses indicate that inactivation of the *TP53* tumor suppressor gene and/or 17p13 deletion represent the most common genetic lesion, observed in ~60% of patients, while disruption of *CDKN2A/B*, a cell cycle regulator, is observed in about 30% of cases (140, 143, 144). Other frequent genetic lesions are represented by *NOTCH1* and *MYC* activating events, present in ~30% of RS cases (62, 140), and are mutually exclusive, consistent with the hypothesis that *NOTCH1* is a transcriptional activator of *MYC* (145). Mutations reported for *NOTCH1* mainly affect the PEST domain, with the P2514fs^*^4 truncating mutation being the most frequent. This event results in the constitutive activation of the pathway as inferred by the presence of the NICD protein and high expression levels of *NOTCH1* target genes (146). However, additional functional studies are needed to understand whether this pathway plays a key role as a transforming event from CLL to RS or if it simply contributes to RS aggressiveness. In addition, a combination of germline genetic characteristics, such as polymorphisms in *BCL2, LRP4* (147) and *CD38* (148) genes, as well as biological features of CLL cells, including unmutated *IGVH* genes, Zap-70 and CD49d expression, represent risk factors for disease development (149).

### Marginal zone lymphoma (MZL)

MZLs, accounting for 5–17% of all non-Hodgkin's lymphomas, derive from B cells of the “marginal zone,” the external part of the secondary lymphoid follicles. The MZ is more evident in the lymphatic tissues continuously exposed to external antigens, such as the mesenteric lymph nodes, the mucosa-associated lymphoid tissues (MALT), and the spleen. MZ B cells act as innate-like lymphocytes able to mount rapid antibody responses mostly to T cell-independent antigens (150).

There are three different MZL entities with specific diagnostic criteria, clinical behavior, and therapeutic implications: the extra-nodal MZL of mucosa-associated lymphoid tissue (MALT) type (MALT lymphoma), the splenic MZL (SMZL), and the nodal MZL (NMZL) (151).

Despite specific alterations peculiar of each subtype, there are genetic lesions and deregulated pathways that are shared (108, 152, 153). Trisomies of chromosomes 3 and 18 and deletion at 6q23 are frequent events in all MZLs, as well as somatic mutations of genes coding for proteins involved in chromatin remodeling (114, 154–156). NF-kB and NOTCH are among the pathways that are recurrently mutated in MZLs, (114, 155, 157–160).

MALT lymphoma is the most common MZL type, accounting for 5–8% of all B cell lymphomas (161). It arises from lymphoid populations that are induced by chronic inflammation in extra-nodal sites. The most frequently affected organ is the stomach, with MALT lymphomas associated to chronic gastritis (162). Beside infections, chronic inflammations caused by autoimmune diseases are risk factors for the development of MALT lymphoma (163). Using targeted sequencing approaches, mutation in *NOTCH1* (8%) and *NOTCH2* (8%) genes have been recently reported (154). Most of the aberrations are frameshift indels and non-sense mutations, clustering in the C-terminal portion of the molecule and specifically in the TAD and PEST domains, in line with mutations and functional effects observed in CLL, MCL, and SMZL. A similar pattern of mutations in *NOTCH2* has been identified through WES/whole-genome sequencing (WGS) studies in SMZL and NMZL, with percentages varying from 10 to 25% in SMZL and ~25% in NMZL (164).

SMZL is a neoplasm of mature B cells that involves spleen, BM, and PB. Within the spleen, tumor cells are represented by small lymphocytes that occupy the MZ surrounding germinal centers and infiltrate the red pulp (123). In SMZL, mutations of *NOTCH2*, together with genes encoding NOTCH signaling pathway components, represent the most recurrent genetic lesions, remarking upon deregulated proliferation and migration as the most affected pathways (114, 165–168). As for MALT lymphomas, most of the identified alterations are frameshift or non-sense mutations clustering within the hotspot region in exon 34, affecting the PEST domain and increasing NOTCH2 activation (114, 169, 170). The 2-bp deletion (p.P2515fs^*^4) in *NOTCH1*, recurrent in CLL (61, 62, 171) and MCL (93), is observed in a minor percentage of SMZL (~5%), (114, 169, 172–174). Furthermore, mutations in *SPEN* and *DTX1* are reported in a small percentage (5%) of SMZL cases (114). The former plays a role in restraining NOTCH signaling through a physical interaction with and consequent repression of the transcription factor RBPJk (165, 175, 176). In physiological conditions, SPEN is as a negative regulator of B lymphocyte differentiation into MZ B cells counteracting the activity of NOTCH (165). Mutations truncate SPEN C-terminal domain, which is involved in the interaction with RBPJk and is critical for NOTCH signaling activation. DTX1 is highly expressed in MZ B cells and may be relevant for the late steps of B lymphocyte differentiation (36, 177). It encodes a RING finger ubiquitin ligase that binds NOTCH family members, modulating their signaling activity. In SMZL, mutations map within two distinct functional domains of DTX1, involved in protein interactions (114). Of note, mutations in genes encoding NOTCH signaling components appear to be largely mutually exclusive with an overall rate of mutations of the pathway in SMZL of ~32%.

NMZL is a rare and indolent B cell tumor that differs from SMZL in terms of pattern of dissemination, being primarily a nodal B cell cancer without clinical evidence of extranodal or splenic disease (123, 178). In general, the mutational profile of NMZL is quite similar to that of SMZL, as revealed by WES studies (179). Specifically, NOTCH signaling pathway appears to be mutated mainly in *NOTCH2* (20%), *SPEN* (11%), and *RBPJL* (6%) (179).

### Multiple myeloma (MM)

In the last decade, several studies have highlighted a role for the NOTCH pathway in MM, a tumor characterized by the proliferation of BM post-GC plasma cells, with release of monoclonal antibodies in blood (180). From the pathological standpoint, MM is characterized by profound genomic instability, epigenetic alterations and strongly dependent on the interaction with the microenvironment (181–184). Indeed, MM cells localization in the BM milieu allows direct interactions between tumor and non-tumor cells residing in the BM, via adhesion molecules and soluble factors, which promote neoplastic cell growth, survival, bone disease, acquisition of drug resistance, and consequent disease relapse. Cumulative evidence indicates a key role of NOTCH signaling in MM onset and progression (185–188).

Unlike other NOTCH-related malignancies, where the majority of patients carry gain-of-function mutations in pathway members, in MM cells NOTCH signaling is aberrantly activated due to increased expression of both receptors and ligands (185, 186). In physiological conditions, hematopoietic stem cells express NOTCH receptors receiving signals from ligands expressed by BM stromal cells. This mechanism contributes to stem cell renewal, survival and differentiation. In MM, this interplay is hijacked by tumor cells to enhance their proliferative rate and escape from therapy (189). Moreover, experimental evidence indicates that NOTCH signaling directly regulates the expression and function of CXCR4, thereby controlling trafficking of MM cells toward BM niches, a mechanism similar to that described in CLL (190).

Several independent studies report the overexpression of NOTCH1 and 2 and the two ligands Jagged1 and Jagged2 by MM cells (185, 186, 191, 192) during disease progression. Indeed, increased NOTCH1 and Jagged1 expression parallels the transition from monoclonal gammopathy of undertermined significance (MGUS) to MM (186), while NOTCH2 expression is increased in more aggressive subsets of patients, carrying specific translocations (193). Of note, most of the genes belonging to the NOTCH signaling pathway are located on chromosomes found to be numerically altered in selected subtypes of MM (9).

Notably, the overexpression of the “NOTCH network” also results in the activation of NOTCH signaling in surrounding stromal cells, contributing to myeloma cell proliferation, survival, and migration, as well as to bone disease and intrinsic and acquired pharmacological resistance. This “backward signaling” occurs as a consequence of activation of NOTCH receptors on BM stromal cells by ligands expressed by MM cells. This interaction results in a NOTCH-mediated upregulation of IL-6 secretion by BM stromal cells, one of the most important growth and survival factors for MM cells (188). The existence of a bidirectional NOTCH signaling offers novel hints in the study of B cell malignancies, and opens new perspectives in the therapeutic scenario.

## Is notch druggable in B cell malignancies?

Independently of the mutational status, NOTCH signaling activation is tightly regulated at multiple steps, thereby providing different strategies to therapeutically target this pathway. In a top-down view, we can envisage at least three levels of intervention: (i) in the extracellular space, interrupting receptor/ligand interactions with specific blocking antibodies; (ii) at the membrane level, preventing the enzymatic cleavages critical for NOTCH activation; and (iii) inside the cells, exploiting selective inhibitors of the “NOTCH interactome.”

Given the widespread involvement of this molecular family in cancer, the NOTCH “drug market” has rapidly grown with the development of antibodies or inhibitors, tested in multiple clinical trials, particularly in the context of solid tumors and T-ALL, with only studies in B cell malignancies (9, 194).

Initial results using γ-secretase inhibitors (GSI) demonstrated excessive toxicity, particularly at the gastrointestinal levels, mainly due on one side to off-target effects and on the other side to the simultaneous targeting of all NOTCH isoforms, expressed on several tissues, thus interfering also with the physiological role of this pathway. The introduction of different schemes of administration, the combination with steroids and the design of more selective inhibitors and drugs have partly overcome these side effects, attaining promising therapeutic responses. In line with the aim of increasing treatment specificity while minimizing toxicity, monoclonal antibodies, specifically binding single members of the NOTCH family, were designed as an alternative approach (195). However, these reagents have not yet reached clinical trials in B-cell malignancies.

An alternative to GSI approaches is immunotherapy, based on the use of blocking antibodies against NOTCH, Delta/Jagged ligands or other extracellular components involved in the NOTCH signaling cascade. Since these drugs affect selectively a NOTCH family member or ligand, they could potentially show fewer side effects compared to GSI (195–198).

Even if limited results have been obtained in clinical trials for B cell neoplasms, promising evidence for the use of these drugs is coming from *in vitro* and *ex-vivo* data obtained in MCL (93) and MM (84). Treatment with GSI resulted in decreased proliferation and increased apoptosis of MCL cell lines, with a concomitant modulation of a selected set of genes strictly dependent on NOTCH. These inhibitors proved to be effective in MM acting directly on tumor cells by enhancing apoptosis but also preventing the BM stroma-mediated protection of MM cells from drug-induced apoptosis. Furthermore, GSI is able to enhance the cytotoxicity induced by selective chemotherapeutic agents, including doxorubicine (85).

Similar effects of GSI, used alone or in combination with fludarabine, were obtained in the high-risk CLL subset of patients carrying a mutated *NOTCH1*. These cells proved to be sensitive to PF-03084014, a non-competitive and reversible GSI, with inhibition of the constitutive activation of the pathway and modulation of apoptosis, as well as migration of leukemic cells (69). Further evidence sustaining a combination strategy to target CLL cells was recently obtained by Secchiero and colleagues who reported the ability of GSI to enhance the anti-leukemic activity of ibrutinib, independently of the mutational status of *NOTCH1* (199).

Considering that, in hematological B cell malignancies, deregulated NOTCH signaling is ancillary to driver aberrant pathways, a combined therapeutic approach may represent a successful way to target tumor cells.

## Concluding remarks

To bear witness of the importance of NOTCH in normal B-cell ontogenesis, mutations in genes belonging to this pathway are invariably found in all mature B cell malignancies. In some instances, the mutational burden insisting on the pathway reaches one third or more of patients, as is the case for MZL or RS. Overall, the emerging picture is that NOTCH signaling is finely tuned in cell- and microenvironment-specific ways. In B cell malignancies, it works primarily as an oncogene, even though functional studies on the mechanisms and consequences of NOTCH signaling are only starting now. Likely, they will allow to improve its therapeutic targeting, also through the design and validation of more selective drugs.

## Author contributions

FA and TV wrote the manuscript and sketched the figures. SD contributed to the writing and editing of the review. All the authors approved the manuscript for its publication.

### Conflict of interest statement

The authors declare that the research was conducted in the absence of any commercial or financial relationships that could be construed as a potential conflict of interest.
